# A Multidisciplinary Approach for Type 2 Allergic Diseases: What Do Biologics Teach Us?

**DOI:** 10.3390/jpm13060941

**Published:** 2023-06-01

**Authors:** Mauro Maniscalco, Aikaterini Detoraki, Giovanni Sarnelli, Maria Nolano, Amato De Paulis, Giuseppe Spadaro, Elena Cantone

**Affiliations:** 1 Department of Clinical Medicine and Surgery, Federico II University, 80138 Naples, Italy; sarnelli@unina.it; 2 Istituti Clinici Scientifici Maugeri IRCCS, Pulmonary Rehabilitation Unit of Telese Terme Institute, 82037 Telese Terme, Italy; 3 Department of Translational Medicine, Federico II University, 80138 Naples, Italy; kate.detoraki@gmail.com (A.D.); giuseppe.spadaro@unina.it (G.S.); 4 Department of Neuroscience, Reproductive and Odontostomatological Sciences-ENT Section, University of Naples Federico II, 80131 Naples, Italy; maria.nolano@unina.it

Patients with atopic/allergic disorders, including atopic dermatitis (AD), allergic rhino-conjunctivitis (AR), chronic rhinosinusitis with/without nasal polyps (CRSwNP/CRSsNP), bronchial asthma, food allergy, and eosinophilic esophagitis (EoE), often share a common genetic background, a type Th2 polarized immune response, and several environmental factors [1,2].

Th2 immunity has evolved to ensure the integrity of the epithelial barrier and to protect against helminths [3]. Although the pathways underlying Th2-driven inflammation can differ and are related to age and ethnicity, they can share some common mediators [1]. The alarmins, namely, thymic stromal lymphopoietin (TSLP), interleukin (IL)-25 and IL-33, are released from injured epidermal and epithelial cells and play pivotal roles in Th2 cell development [4]. Other cytokines, including IL-4, IL-5, IL-9, IL-13, and IL-31, are also relevant in orchestrating a Th2-type immune response [3]. Naïve CD4^+^ T cells differentiate into Th2 cells under the influence of IL-4, which also stimulates isotype class switching of B-cells in order to produce immunoglobulin E (IgE) [5]. The additional signaling provided by IL-5 and IL-9 leads to eosinophil and mast cell recruitment to the inflammatory site, while IL-13 induces goblet cell hyperplasia, mucus hypersecretion, and airway hyperresponsiveness [6].

Atopic/allergic disorders affect different body systems and, in some cases, coexist simultaneously in the same patient. Conversely, in other cases, there is a progressive and predictable succession in the involvement of other systems (atopic march), with the progressive onset of AD, food allergy, AR, and allergic asthma [7]. Accordingly, asthma is diagnosed in up to 65% and up to 49.8% of patients with CRSwNP and AD, respectively [8]. Furthermore, some data seem to indicate the association of AD with the development of CRSwNP [9]. Asthma and EoE also frequently coexist and are both characterized by eosinophilic inflammation of the mucosa and submucosa [10]. Finally, recent studies have reported AD prevalence rates of between 7% and 55% among EoE patients [11].

When coexisting in the same patient, the severity of asthma, CRSwNP and AD is greater. In such instances, each of them should be treated concurrently to obtain the optimal treatment results [8,12].

The presence of several allergic disorders in the same individuals suggests that some subgroups of allergic diseases share the same subtype of health condition, namely the “endotype” variety, especially the Th2-driven endotype. Indeed, Th2-high inflammation is a feature of allergic asthma, AR, and CRSwNP [13]. A Th2-high, as opposed to Th2-low, immune response has been suggested for the endotyping of AD [13,14,15] and EoE [14,15]. A Th2-high profile has been identified in some types of food allergy and oral allergy syndrome [16].

Recently, new data have indicated that Th2 effector cells are not homogeneous. Indeed, the scientific community has identified a subgroup of pathogenic Th2 cells (Tpath2), which express large amounts of chemoattractant receptor-homologous molecules that are expressed on Th2 cells (CRTH2), CD49d, and CD161. Additionally, researchers have come to identify alarmins, which also produce large amounts of IL-5 and IL-9 [17]. In this regard, IL-33 seems to be required to obtain the optimal effector function of human Tpath2 cells [17], which exacerbate eosinophilic inflammation [18] through the production of a large quantity of IL-5 [19]. The identification of Tpath2 cells has improved the understanding of the pathogenesis of atopic/allergic diseases [20].

In line with a growing body of evidence, if some Th2-driven pathophysiological mechanisms are shared across multiple atopic/allergic disorders, it is possible to hypothesize that, by treating a single clinical condition in an individual with multiple Th2 diseases, a simultaneous improvement can be obtained. Recently the increasing availability of new biological drugs that possess the capacity to interfere, whether directly and indirectly, with the action of single cytokines has allowed effective treatment for several Th2 diseases and related comorbidities.

Omalizumab, a monoclonal antibody targeting the high-affinity receptor binding site of IgE, has demonstrated clinical efficacy in subjects with CRSwNP [21], food allergies [22], severe allergic asthma [23], and moderate-to-severe chronic idiopathic urticaria [24]. Although IgE seems to be implicated in AD, its targeting is clinically ineffective [25], mepolizumab, a humanized monoclonal antibody capable of directly binding free IL-5, has shown clinical efficacy in treating patients with CRSwNP, although its use is ineffective in patients with AD [26]. By contrast, the anti-IL-5Rα Benralizumab has shown remarkable therapeutic effects in various types of eosinophilic diseases, including eosinophilic CRS and asthma [27]. Tralokinumab, a monoclonal antibody inhibiting IL-13, is relatively effective in patients with AD, but not in asthmatic subjects, suggesting that IL-13 is clinically relevant in AD but that its role in the pathophysiology of asthma is smaller [3]. Dupilumab, a humanized monoclonal antibody inhibiting both IL-4 and IL-13 signaling, has been approved for patients with Th2-driven inflammatory disorders, including AD [28], asthma [29] and CRSwNP [30], supporting the key pathogenic role of IL-4 and IL-13 in these conditions. Similar and encouraging results are also emerging in the management of EoE [31]. Finally, the use of Tezepelumab, which targets TSLP, is clinically useful in asthma but has not produced clinically relevant results in AD [3].

The use of biologics to treat severe uncontrolled CRSwNP is quite recent compared to their use in cases of asthma and atopic dermatitis. In fact, there are still few real-life studies, and those in existence are limited to discussions of dupilumab [32]. Furthermore, there are no comparative studies evaluating the indication of one biologic agent versus another for the treatment of different CRS endotypes.

The clinical results obtained from biological drugs directed against single receptors and/or mediators in Th2 inflammatory diseases have allowed researchers to better define their pathogenetic role in each of them. Conversely, and on the other hand, has stimulated the research inro additional biologics directed against other relevant cytokines. However, although the positive clinical response to biologics confirms the association between Th2-driven diseases, no biomarker with high specificity can anticipate the therapeutic response in either single or multiple Th2 conditions.

Although Th2-driven immune responses are multifaceted, the use of biologics has allowed significant therapeutic advances and has clarified that the underlying pathophysiological mechanisms are substantially limited in number. However, many issues still remain to be adequately addressed.

In the context of precision medicine and individualized approaches, future research should focus on the identification of biomarkers that can accurately indicate the specific endotype for a specific patient and, therefore, the most suitable biological drug. Furthermore, it is important to establish the correct timing for the administration of biologics in the clinical course of a Th2-driven disorder. This matter is far from insignificant. In fact, considering that allergic/atopic disorders often coexist and/or follow each other chronologically, it is essential to know whether their early use for the treatment of one of these conditions possesses the capacity to prevent “atopic march”.
